# Modeling the Geographic Consequence and Pattern of Dengue Fever Transmission in Thailand

**Published:** 2016-05-04

**Authors:** Collins Bekoe, Tatdow Pansombut, Pakwan Riyapan, Sampurna Kakchapati, Aniruth Phon-On

**Affiliations:** ^1^ Department of Mathematics and Computer Science, Faculty of Science and Technology, Prince of Songkla University, Pattani campus, Pattani, Thailand

**Keywords:** Infectious Disease, Dengue, Linear Models, Thailand

## Abstract

**Background:** Dengue fever is one of the infectious diseases that is still a public health problem in Thailand. This study considers in detail, the geographic consequence, seasonal and pattern of dengue fever transmission among the 76 provinces of Thailand from 2003 to 2015.

**Study Design:** A cross-sectional study.

**Methods:** The data for the study was from the Department of Disease Control under the Bureau of Epidemiology, Thailand. The quarterly effects and location on the transmission of dengue was modeled using an alternative additive log-linear model.

**Results:** The model fitted well as illustrated by the residual plots and the R^2^ (0.49). Again, the model showed that dengue fever is high in the second quarter of every year from May to August. There was an evidence of an increase in the trend of dengue annually from 2003 to 2015.

**Conclusions:** There was a difference in the distribution of dengue fever within and between provinces. The areas of high risks were the central and southern regions of Thailand. The log-linear model provided a simple medium of modeling dengue fever transmission. The results are very important in the geographic distribution of dengue fever patterns.

## Introduction

Dengue fever, a debilitating viral infection among the major infectious diseases has grown spectacularly in recent times with a rapid transmission globally. Dengue fever is a vector-borne disease and the female *Aedes agypti* is the principal carrier of the virus^1^. The symptoms of dengue range from mild to high fever with intense headache and body pains^1^.

According to WHO, there is no specific cure for dengue and that fatality rate can only be reduced with an early detection and good medical care. There has been several clinical trials over the last 50 years aiming at assessing therapeutic options but to no major successes^2, 3^. As a result of the difficulties and lack of a specific drug to cure the disease, much more attention has been given to the prevention of the disease by resorting to various vector control measures. The vector control measures include physical (destruction of breeding sites), biological (using bacteria like *Bacillus thuringiensis*) and chemical ( insecticides) means to control the vector population^4^. An estimate by WHO places almost half of the total number of people worldwide at risk of getting dengue fever disease^6^. About 3.9 billion people live in dengue prone areas in 128 countries^5^ with almost 390 million of the cases identified clinically^6^.

Dengue fever is still a health menace in the southeastern part of Asia and is one of the top priorities among major infectious diseases in Thailand^7^. From 1985 to 1999, there were 69,000 cases per year on the average in Thailand^8^. Thailand was also the sixth highest country among 30 most highly endemic countries in the world from 2004 to 2010 with an average of 74,292 cases and 83 deaths according to WHO^8^. Thailand’s National disease surveillance report indicated that a total of 30,108 cases had been reported in all provinces from January 2016 to October 2016 with 4 deaths. The morbidity rate was 0.01/100,000 population^9^.

Every country requires its public health officials to always evaluate the extent to which any disease outbreak would have on the country. The characterisation of the trend of diseseases and its associated changes is of great importance in evaluating the success of the control measures, health planning and health development schemes^10^. Health professionals take into consideration the disease status within a particular area, the time frame and those at risk in order to inform the appropriate actions. Being able to understand the risk factors and also the spatial and temporal distribution of the disease is important for proper public health interventions. The necessary quantitative framework for analyzing and understanding vital issues in conjunction with the disease can be done through statistical modelling. Investigating and understanding the transmission patterns and also the ability to predict disease outbreaks can be done using statistical models. The models can bring out essential details pertaining to the prevalence of the disease, mortality rates and prediction of the health status of the population over a certain period of time. Statistical models have been applied in the study of infectious diseases such as tuberculosis in Nepal and Thailand and also malaria in Nepal^11, 12, 13^. The models highlight these issues which become a blueprint for public health professionals to make good use of available epidemiological data. A critical tool for dengue fever monitoring, control and prevention is the ability to predict its seasonality, risk and occurrences^14^. Again, an accurate forecast of the incidence rate can provide a way to efficiently apply public health programs to prevent and control diseases^15^. The study therefore aims to examine the regional distribution of dengue in Thailand in order to highlight areas of high risk for proper attention to be given in an untimely outbreak of the disease.

## Methods

This study presents a retrospective insight of the number of cases of dengue fever from Thailand’s National Disease Surveillance report^9^. Thailand’s National disease surveillance system has different dataset on dengue fever, dengue hemorrhagic fever and dengue shock syndrome. However, the study used the data on the dengue fever since the dengue hemorrhagic fever and the dengue shock syndrome are the extreme forms of dengue fever. The study defines dengue fever case as an individual who has been reported sick and is showing signs and symptoms of the disease as reported by the hospitals and health centers. The Thailand’s National Disease Surveillance System is under the Bureau of Epidemiology, Department of Disease Control, Ministry of Public Health. The reporting system records diseases from provincial public health offices like hospitals and health centers in Thailand. The data obtained spans 13 yr from 2003 to 2015 from all 76 provinces in Thailand. The reporting system of dengue fever disease during the 13-year period has basically been the information the hospitlas and the health centers provide on each patient across Thailand^16^. The study defines the number of cases as the number of people who report sick at the various hospitals and health centers by that system. The dataset consisted of reported cases and deaths in Microsoft Word file by province and by month with all the provinces arranged into four regions. The regions were the north, central, north-east and the southern region. The file was then converted to a spreadsheet. The fields in the spreadsheet contained the reporting areas, in this case the provinces and the cases recorded for each month beginning from January to December for each year. The provinces were arranged with a unique identification number starting from 1 to 76. Table 1 shows the list of all the 76 provinces in Thailand and their identification numbers.

**Table 1:** Provinces in Thailand and their respective Id’s

**ID**	**Province**	**ID**	**Province**	**ID**	**Province**
1	Chiang Mai	27	Kanchanaburi	53	Maha Sarakham
2	Chiang Rai	28	Nakhon Pathom	54	Roi Et
3	Lampang	29	Ratchaburi	55	Buri Ram
4	Lamphun	30	Suphan Buri	56	Chaiyaphum
5	Mae Hong Son	31	Phetchaburi	57	Nakhon Ratchasima
6	Nan	32	Prachuap Khiri Khan	58	Surin
7	Phayao	33	Samut Sakhon	59	Amnat Charoen
8	Phrae	34	Samut Songkhram	60	Si Sa Ket
9	Phetchabun	35	Chachoengsao	61	Ubon Ratchathani
10	Phitsanulok	36	Nakhon Nayok	62	Yasothon
11	Sukhothai	37	Prachin Buri	63	Chumphon
12	Tak	38	Sa Kaeo	64	Ranong
13	Uttaradit	39	Samut Prakan	65	Surat Thani
14	Kamphaeng Phet	40	Chanthaburi	66	Nakhon Si Thammarat
15	Nakhon Sawan	41	Chon Buri	67	Phatthalung
16	Phichit	42	Rayong	68	Trang
17	Uthai Thani	43	Trat	69	Krabi
18	Bangkok	44	Loei	70	Phangnga
19	Ang Thong	45	Nong Bua Lam Phu	71	Phuket
20	Nonthaburi	46	Nong Khai	72	Narathiwat
21	P. Nakhon S. Ayutthaya	47	Udon Thani	73	Pattani
22	Pathum Thani	48	Kalasin	74	Yala
23	Chai Nat	49	Mukdahan	75	Satun
24	Lop Buri	50	Nakhon Phanom	76	Songkhla
25	Saraburi	51	Sakon Nakhon		
26	Sing Buri	52	Khon Kaen		

The explanatory variables for investigating the occurrence rates of dengue fever were residential area (by province), quarter of the year and year with the year grouped into three quarter periods with January to April, May to August and September to December. The categorisation is as a result of the fact that weather variability has over the years been identified as a factor for the increase in the number of reported cases at certain times in the year^17^. Therefore, the study sorted to identify from the data the time period that dengue fever peaks the most in the year as a result of the rainy season.

### Statistical Methods

The statistical method employed in the study required that dengue fever cases in cells defined by province x, quarterly y and z represents the magnitude of the number of cases reported n_xyz_ to p_x_, with its associated population at risk in 1000s. The occurrence rates with normally distributed errors was calculated using an alternative additive log-linear model^18^ which is

(1) $$In\left(\frac{n_{xyz}}{P_{x}}\right)=\gamma_{jqt}=\mu +\alpha_{x}+\beta_{y}+\gamma_{z}$$

From equation (1), *α _x_ , β_y_*and *ɣ_z_*represent province, quarterly and year effects respectively that add up to zero with *μ* as a constant that put together the overall incidence. Normally when data from the field on epidemics are recorded, some cells with no reported cases are recorded as zero and this does not allow for log-transformation. As a result, they are replaced by a small constant *k*, but the values for *n_xyz_* greater than zero are maintained. The standardized residuals were plotted against the normal quantiles as an assessment of the model. Again based on the model, the observed counts and the number of reported cases are plotted against its associated fitted values. R-squared was then used in order to check the model’s ability in measuring the variations in the data. The following formula was used to obtain the estimates of the case rate.

**Figure:**
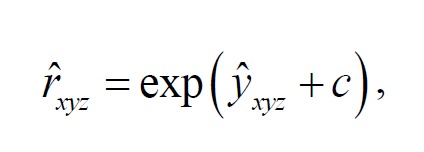


From the above equation, ${\hat{y}}_{xyz}$ represents *y_xyz_* , its fitted value with a constant *c* for equaling the overall observed dengue cases from the model.

Dengue fever case rates for each level of each variable of interest adjusted for other variables was computed after the model was fitted. Sum contrasts was then used to obtain the standard errors for the adjusted dengue occurrence rates. It was then compared with the overall mean of the dengue fever transmission rate. As a result, the transmission pattern of dengue fever was keyed out for each variable of interest. Normally, confidence intervals for variable-specific dengue fever cases obtained by the above method naturally separates into three groups. The division is done according to the position on the mean line. That is whether they are entirely above, below or on the mean line. This three–part division–result was then used to create a thematic map for all the provinces in accordance with their dengue fever annual incidences. In this study, the R program (version 3.3.1) was used for all the statistical analysis and also for plotting the graphs and maps^19^.

## Results

During the 13-yr period as shown from the data obtained, the Bureau of Epidemiology has recorded a total of 468,234 dengue fever cases from 2003 to 2015. There has been 110,946 number of cases from the North, 162,120 number cases from the central, 121,702 number of cases from the North east and 73,466 number of cases from the southern part of Thailand.

### Statistical Analysis

**Figure 1:**
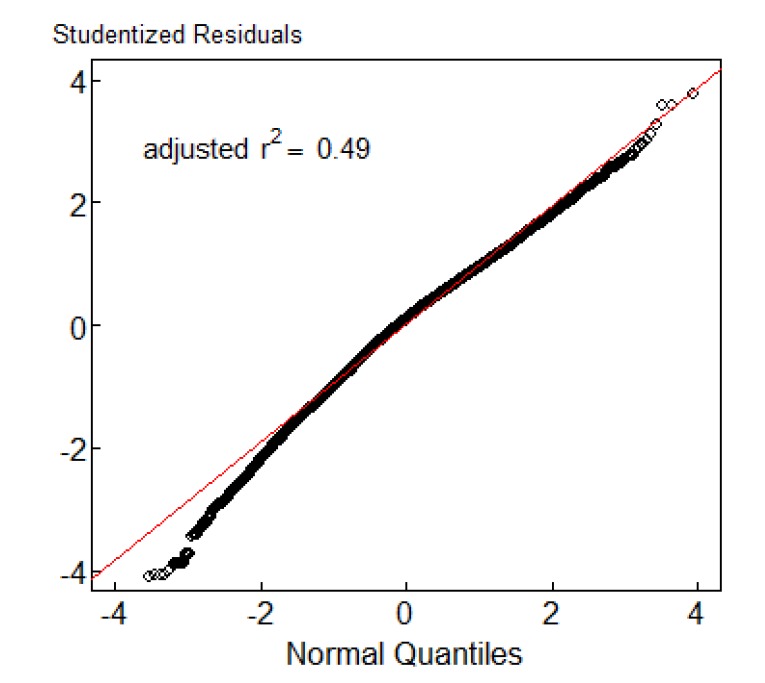
Diagnostic residual plots for log-normal for Dengue fever cases in Thailand

Figure 1 shows the results of the model fitting with deviance residuals plotted against the normal quantiles using the alternative additive log-linear model from equation (1) replacing the zero counts with one. It shows that the residuals plot from the linear model on the log-transformed number of reported cases fit the data well.

**Figure 2:**
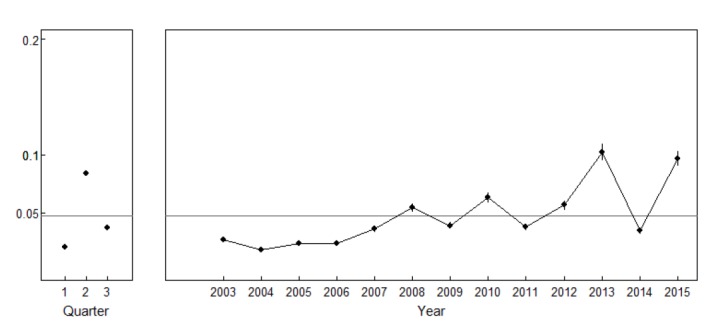
95% confidence intervals of the annual reported cases in Thailand by quarter and year

Figure 2 shows 95% confidence intervals of the annual reported cases by quarter and year. The mean incidence of dengue fever was 0.047/1000 population. Based on the log linear model, the number of reported cases by provinces has been conformed to the outcome of the rest of the variables in equation (1). The horizontal lines from the figure represent the average number of reported cases (0.047 per 1000). From the graph, dengue fever was high in the second quarter from May to August and there was an increase in the reported cases from 2003 to 2013, then followed by a drop in 2014 and then an increase in 2015.

**Figure 3:**
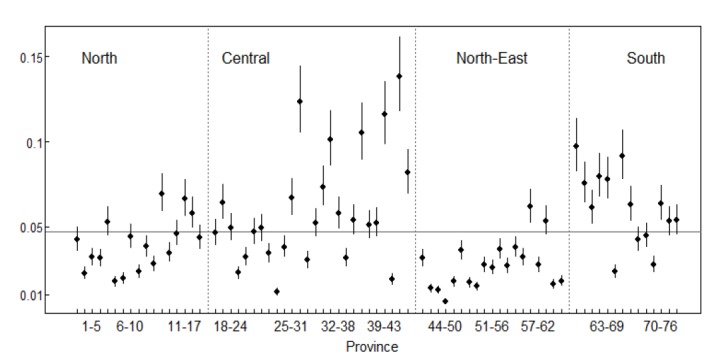
Dengue cases/1000 by province

Figure 3 shows the 95% confidence intervals representing the annual reported cases by province. All the 76 provinces in Thailand were divided into North, Central, Northeast and South using the dotted vertical lines.

Provinces with confidence intervals above the mean were grouped as having a higher than average incidence rate, provinces with confidence intervals below the mean were grouped as having a lower than average incidence rate and finally provinces with confidence intervals overlapping the mean were categorized as having an average incidence.

**Figure 4:**
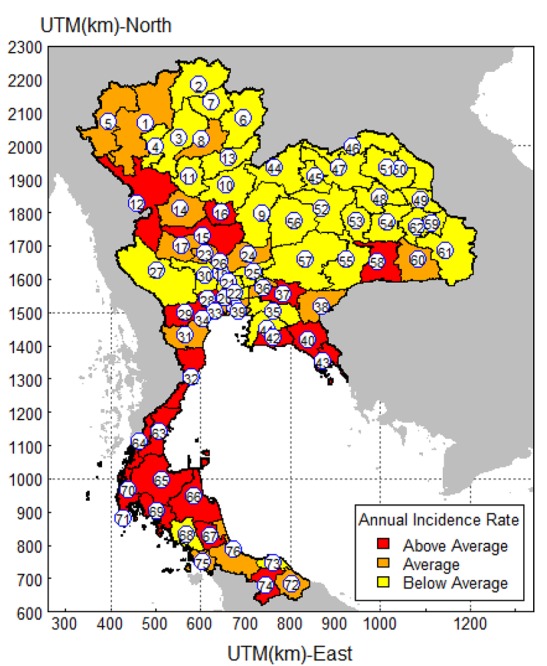
Thematic map showing the distribution of Dengue fever incidence in Thailand

The above statistical analysis provides a blueprint in understanding and knowing graphically how dengue fever has been spreading in Thailand during the period under review. Figure 4 therefore presents a detailed thematic map showing the adjusted annual reported cases by provinces. This was done by making use of the confidence intervals shown in Figure 3 by putting the provinces as above (red color), below (yellow color) or close to the mean line (orange color). The following provinces had a much higher dengue incidence as compared with the mean line. These provinces are Ratchaburi, Samut Sakhon, Prachin Buri, Chanthaburi and Rayong. Chumphon, Ranong, Nakhon Si Thammarat, Phatthalung and Krabi provinces also recorded cases that were more than the average number of reported cases.

## Discussion

In this study, linear regression models comprising of quarter of the year and provinces as determinants were fitted to the log-transformed number of dengue fever cases replacing zero cells counts with a constant before log-transformation. The adjusted R-squared from the model was 0.49 and this means that the model fitted well. However, the overall annual reported cases from all the 76 provinces of Thailand were 0.0047.

During the 13-yr period of the current study (2003-2015), there was increasing trends of the occurrences of dengue fever. The transmission trend shows dengue fever increasing from 2003 to 2008 with a drop in 2009 and an increase in 2010. The reported cases dropped again in 2011, which then rose through 2012 to 2013. There was a sharp drop in 2014, which was followed by another sharp increase in 2015. However, during each second quarter (May, June, July, and August); the incidence rates were higher with much lower rates found during the first and third quarter of each year. This finding is consistent with studies done on trends and patterns of dengue fever in Thailand^7, 20-23^. This is probably because of the changes in the climate^24^ and the connection between the vectors and the rainy months. The pattern realized concurs with the rainy season in Thailand, which sometimes is different from province to province and region to region. The Thailand Meteorological Department has shown that the rainy season is from mid-May to mid-October every year^25^. The role of temperature, precipitation and humidity are important environmental drivers that influence dengue fever transmission^14^. However, the possibility of rainfall and temperature playing a role in the transmission dynamics of dengue in Thailand was not investigated in the study.

The study has also highlighted the high occurrences of dengue in Ratchaburi, Samut Sakhon, Prachin Buri, Chanthaburi and Rayong provinces. All these provinces are in the central region of Thailand. Again, Chumphon, Ranong, Nakhon Si Thammarat, Phatthalung and Krabi provinces in the southern part of Thailand also had higher than average incidence rates. However, the Northeastern part of Thailand recorded a much lower incidence of dengue with the North having an average number of reported cases. Urban migration and perhaps cross-border population movement can be contributing factors for the transmission of dengue in these parts of Thailand.

The major limitation of this study however is that the actual dengue fever incidence in Thailand is not sharply exact as the surveillance data collected by the Bureau of Epidemiology among other infectious diseases is known to be under-recorded^26-28^. On the other hand, the absolute extent of the incidence is inaccurate; however, the findings from this study should depict the relative transmission pattern of dengue fever occurrences among all the provinces in Thailand.

There were a few limitations in the study as it was based on secondary data. In addition, various risks factors were not included due to unavailability of data. Further analyses in the future are required in order to appraise the trends of dengue fever disease with data that spans over a long period.

## Conclusions

Dengue fever remains a public health problem in Thailand, as the disease is present in all the provinces of the kingdom. Various interventions at the national, district levels and improvements in health services should be continued to control the high burden of dengue fever. Moreover, the recent increase (2015) in the incidence of dengue fever needs to be investigated by further research. The map that shows the provinces with higher incidence rates illustrates the findings. Knowledge with areas of high risk using such maps can help public health authorities to prioritize preventive measures to control subsequent dengue fever outbreaks. The health officials can focus on zones with a high or increasing dengue fever. The study therefore recommends intensifying the education on dengue fever incidence to sensitize people in the provinces of its existence and then entreating them to sleep under treated mosquito nets. Research into producing a more effective drug for vaccination against dengue fever should be intensified in order to protect people against the disease. Lastly the government and other policy makers should increase the control measures like the physical (destruction of breeding sites), chemical (application of insecticides) and biological (use of bacteria like *Bacillus thuringiensis*) means to target the vector population in order to reduce the transmission of the disease.

## Acknowledgements

We would like to show our appreciation to the higher education research promotion and the Thailand Education Hub for Southern Region of ASEAN Countries Project Office of the Higher Education Commission.

## Conflict of interest statement

The authors declare no conflict of interest.

## Funding

This work was supported by the Higher Education Research Promotion and the Thailand’s Education Hub for Southern Region of ASEAN Countries Project Office of the Higher Education Commission.

## Highlights

- Dengue fever incidence rate is very high from May to August every year in Thailand.
- Dengue fever incidence is high in the central and southern regions of Thailand.
- The thematic map provides a blueprint for proper public health interventions.
